# Engineering monosex and sterile fish for food production: from conventional methods to CRISPR-based precision

**DOI:** 10.1007/s44307-026-00128-5

**Published:** 2026-08-04

**Authors:** Yue Min, Fei Sun, Joey Wong, May Lee, Gen Hua Yue

**Affiliations:** Temasek Life Sciences Laboratory, 1 Research Link, National University of Singapore, Singapore, SG 117604 Singapore

**Keywords:** Genome editing, Germ cell transplantation, Monosex production, Reproductive biocontainment, Sustainable aquaculture

## Abstract

Monosex and sterile fish populations are increasingly recognized as essential tools for enhancing aquaculture productivity while safeguarding ecosystems from genetic introgression and uncontrolled reproduction. Conventional approaches such as hormonal manipulation and triploidy have demonstrated utility but face persistent limitations in efficiency, welfare, and regulatory acceptance. Recent breakthroughs in CRISPR-based genome editing and germ cell transplantation now enable heritable, scalable, and biocontained control of sex and fertility, offering transformative potential for sustainable aquaculture. This paper synthesizes advances across finfish and crustaceans, critically evaluates technical and regulatory bottlenecks, and highlights convergent solutions integrating automation, artificial intelligence, and governance frameworks. By positioning reproductive programming as a cornerstone of climate-resilient and sustainable aquatic food systems, we propose a roadmap toward commercial adoption that balances biological precision, ecological safety, and societal acceptance.

**Supplementary Information:**

The online version contains supplementary material available at 10.1007/s44307-026-00128-5.

## Introduction

As the demand for aquaculture products continues to rise, the industry faces challenges in meeting sustainable and efficient production goals (Naylor et al. 2021). A critical aspect is controlling fish reproduction and improving growth, in which producing monosex and sterile populations has shown significant benefits (Hindar et al. 2023; Basavaraju 2023; Xu et al. 2024). For example, all-male Nile tilapia populations are widely used because males generally grow faster than females (Beardmore et al. 2001), while monosex culture reduces early maturation, prevents uncontrolled reproduction, and minimizes size variation (Borges et al. 2005; Luckenbach et al. 2017; Wahl et al. 2025). Monosex populations are desirable in species with distinct sexual growth advantages, as they can reduce issues like early maturation and unwanted reproduction (Basavaraju 2023). Similarly, sterile triploid salmonids, such as Atlantic salmon and rainbow trout, have been applied or evaluated to reduce pre-harvest sexual maturation and limit genetic introgression from escaped farmed fish into wild populations (Benfey 2001; Weber et al. 2014). Triploid grass carp (Zajicek et al. 2011) also represent a classical example in which sterility is used for biological containment. When stocked for aquatic vegetation control, sterile fish minimize ecological risks associated with escape (Hindar et al. 2023). Taken together, these cases demonstrate that control of sex and fertility is emerging as a foundational biotechnology for sustainable aquaculture, analogous to hybrid seed systems in terrestrial agriculture (Thakur et al. 2026).

Sexual size dimorphism (SSD) significantly affects aquaculture efficiency and profitability (Wan et al. 2021). Monosex production offers advantages: preventing premature maturation, optimizing growth rate, enhancing product quality, and mitigating environmental risks (Basavaraju 2023). Several conventional approaches have been used: (1) Hormonal sex reversal using steroids (Chu et al. 2023); (2) Aromatase inhibitors and thermal treatments (Xiong et al. 2022); (3) Gynogenesis and androgenesis (Komen & Thorgaard 2007); (4) Polyploidy manipulation to create sterile triploids (Piferrer et al. 2009); (5) Sex-reversal-assisted genetic breeding for producing supermales (Chen et al. 2019); and (6) Surgery and RNAi (RNA interference) in crustaceans (Fowler & Leonard 1999; Wahl et al. 2023). While improving productivity, these methods vary in feasibility, cost, efficiency, and raise environmental or ethical concerns. Over the past decade, the convergence of CRISPR-based genome editing (GE) (Hallerman 2021; Huang et al. 2023), germ cell transplantation (GCT) (Kleppe et al. 2025), and reproductive genomics (Zhai et al. 2022) has enabled heritable, scalable, and biocontained control of fish sex and fertility (Huang et al. 2023). Sex-controlled breeding in aquaculture has also relied on several conventional approaches, including hormone-induced sex reversal (Abo-Al-Ela 2018), marker-assisted selection (Yue 2014), and triploidy (Komen & Thorgaard 2007), which have already contributed to improved production efficiency in some species. However, these methods may involve long breeding cycles, high operational costs, incomplete stability, or concerns related to welfare and environmental safety. Unlike triploidy, which induces sterility through chromosomal imbalance (Benfey 2016), GE can disrupt individual genes (e.g., *dmrt1*, *dnd*, *iag*) to produce fully diploid, reproductively sterile or monosex fish with near-wild-type performance. When combined with surrogate broodstock technology (Jin et al. 2021; Nagasawa et al. 2019), the gene edits are confined to the recipient animals, which act as “living bioreactors”. They are made sterile and then implanted with elite donor germ cells. Consequently, they produce offspring that inherit the donor’s genome exclusively. Nevertheless, despite their technical advantages, GE- and GCT-based strategies remain difficult to implement at commercial scale (Nagasawa et al. 2019; Wang et al. 2025). Their successful application is currently limited to a relatively small number of model or economically important fish species, and their efficiency, delivery systems, germline competence, regulatory acceptance, and cost-effectiveness remain major bottlenecks for many aquatic organisms (Hallerman et al. 2024; Robinson et al. 2024; Wang et al. 2025; Huang et al. 2023). This paper examines how these tools are transforming aquaculture from a phenotype-driven to a programmable enterprise. We synthesize breakthroughs in monosex and sterility engineering across finfish and crustaceans, critically evaluate bottlenecks in efficiency and regulation, and propose a convergent roadmap, integrating AI, automation, and global policy, to accelerate commercial adoption. The goal is not just faster-growing fish, but climate-resilient, ecologically contained food systems built on precise biological control.

## Advantages of monosex and sterile fish production in aquaculture

In many fish species, sex strongly influences market value due to growth rate and size differences (Beato et al. 2026). For example, male giant shell dweller (*Lamprologus callipterus*) weighs over 12 times more than females (Schutz & Taborsky 2000). In Nile tilapia (*Oreochromis niloticus*), males grow up to 40% larger than females (Nkanta et al. 2025), while yellow catfish (*Pelteobagrus fulvidraco*) also shows male-biased growth, with males exhibiting a markedly faster growth rate than females. Male-biased SSD is also observed in salmonids (Morita & Sato 2025) and prawns (*Macrobrachium* spp.) (Irving et al. 2017). Conversely, female-biased SSD occurs in species like half-smooth tongue sole (*Cynoglossus semilaevis*), where females reach four times the size of males (Beato et al. 2026). In Atlantic cod (*Gadus morhua*), males are ~ 10% smaller than females (Gardiner et al. 2025).

As demonstrated in the previous section, monosex populations confer significant advantages (Basavaraju 2023; Beato et al. 2026). Several approaches have been used to establish unisexual populations in fish, including steroid hormone-induced sex reversal (Huang et al. 2023; Pandian & Sheela 1995), parthenogenesis-related (Zhou et al. 2000), chromosome-set manipulation (Komen & Thorgaard 2007), and genetic breeding strategies (Chen et al. 2019; Mair et al. 1995). Steroid hormone induction generally involves the administration of exogenous androgens or estrogens during the labile period of sex differentiation to direct gonadal development toward the desired phenotypic sex (Pandian & Sheela 1995; Huang et al. 2023). For example, androgens such as 17α-methyltestosterone are commonly used for masculinization, whereas estrogens such as estradiol-17β can be used for feminization (Pandian & Sheela 1995; Mondal et al. 2025). These hormones are usually administered through treated feed or immersion, depending on the species and developmental stage (Mondal et al. 2025). Parthenogenesis-related methods, such as gynogenesis and androgenesis, use maternal or paternal genomes to generate offspring with specific genetic backgrounds and sex chromosome compositions (Komen & Thorgaard 2007). In addition to these direct sex-control methods, genetic breeding strategies have also been widely used. A representative example is YY “supermale” technology in tilapia, in which YY males are produced through crosses involving sex-reversed XY females and normal XY males; these YY males can then be crossed with XX females to generate predominantly all-male offspring (Liu et al. 2026; Mair et al. 1995).

Sterile fish improve productivity and sustainability (Hindar et al. 2023; Wang et al. 2025). They prevent unwanted reproduction, ensuring consistent growth and efficient feed conversion, reduce resource competition and environmental impact, and minimize genetic contamination of wild populations. Sterility can also enhance growth by redirecting energy from reproduction to somatic development. For example, rainbow trout (*Oncorhynchus mykiss*) and Atlantic salmon (*Salmo salar*) are often triploidized via thermal or pressure shocks to induce sterility (Kuciński et al. 2024). This renders them functionally sterile and may improve growth under some conditions while reducing the risk of genetic contamination. However, conventional triploidy-based sterility is not always welfare-neutral, as triploid fish in some species have been associated with cataracts, skeletal deformities, or other developmental abnormalities (Fraser et al. 2012). Although triploids are functionally sterile, residual gonadal development and reduced welfare under suboptimal conditions (Fraser et al. 2012; Benfey 2016) have raised concerns regarding robustness and performance. GE (Hallerman 2021; Yang et al. 2022; Wang et al. 2025) combined with GCT (Yoshizaki & Yazawa 2019; Okutsu et al. 2006) has the potential to overcome the limitations of conventional methods for producing monosex and sterile food fish, while substantially accelerating the efficiency and speed of the entire process.

## New approaches for producing monosex and sterile fish: GE and GCT

CRISPR/Cas systems for GE have revolutionized biological research with broad applications in aquaculture (Hallerman 2021; Yang et al. 2022). The technology utilizes Cas proteins (e.g., Cas9) as molecular scissors and a guide RNA to direct DNA cleavage at specific sequences (Cong et al. 2013). Variants like Cas12a offer staggered cuts (Swarts & Jinek 2018), while Cas13 targets RNA (Abudayyeh et al. 2017). Engineered proteins like dCas9 enable gene regulation without cutting (Xu & Qi 2019). Base editing allows single-nucleotide changes without double-strand breaks (Rees & Liu 2018), and prime editing enables more versatile modifications (Chen & Liu 2023). Compared with conventional double-strand break–dependent editing, base and prime editing markedly reduce unintended insertions/deletions and off-target effects (Chen & Liu 2023), thereby improving editing precision and genomic stability. These next-generation tools also expand the editable sequence space and allow predictable, programmable point mutations, insertions, and corrections without requiring donor templates. Such advantages make these tools particularly well suited for aquaculture applications. In fish, it enables targeted modification of genes related to sex determination, fertility, and germ-cell development. Recent advances, including base editing (Rees & Liu 2018) and prime editing (Chen & Liu 2023), further improve editing precision and expand the range of editable targets, making these tools useful for fine-tuning economically important traits and functional genomics studies in aquaculture species (Wang et al. 2025). GE in fish is typically achieved by delivering CRISPR/Cas or other genome editors into embryos or germ cells (Robinson et al. 2024; Yang et al. 2022). Traditional microinjection directly introduces reagents into fertilized eggs but is labour-intensive. Emerging approaches include electroporation, viral vectors, and nanoparticle-mediated delivery, offering less invasive, higher-throughput, and scalable alternatives suitable for industrial aquaculture applications (Chen & Liu 2023; Yang et al. 2022).

**Fig. 1:**
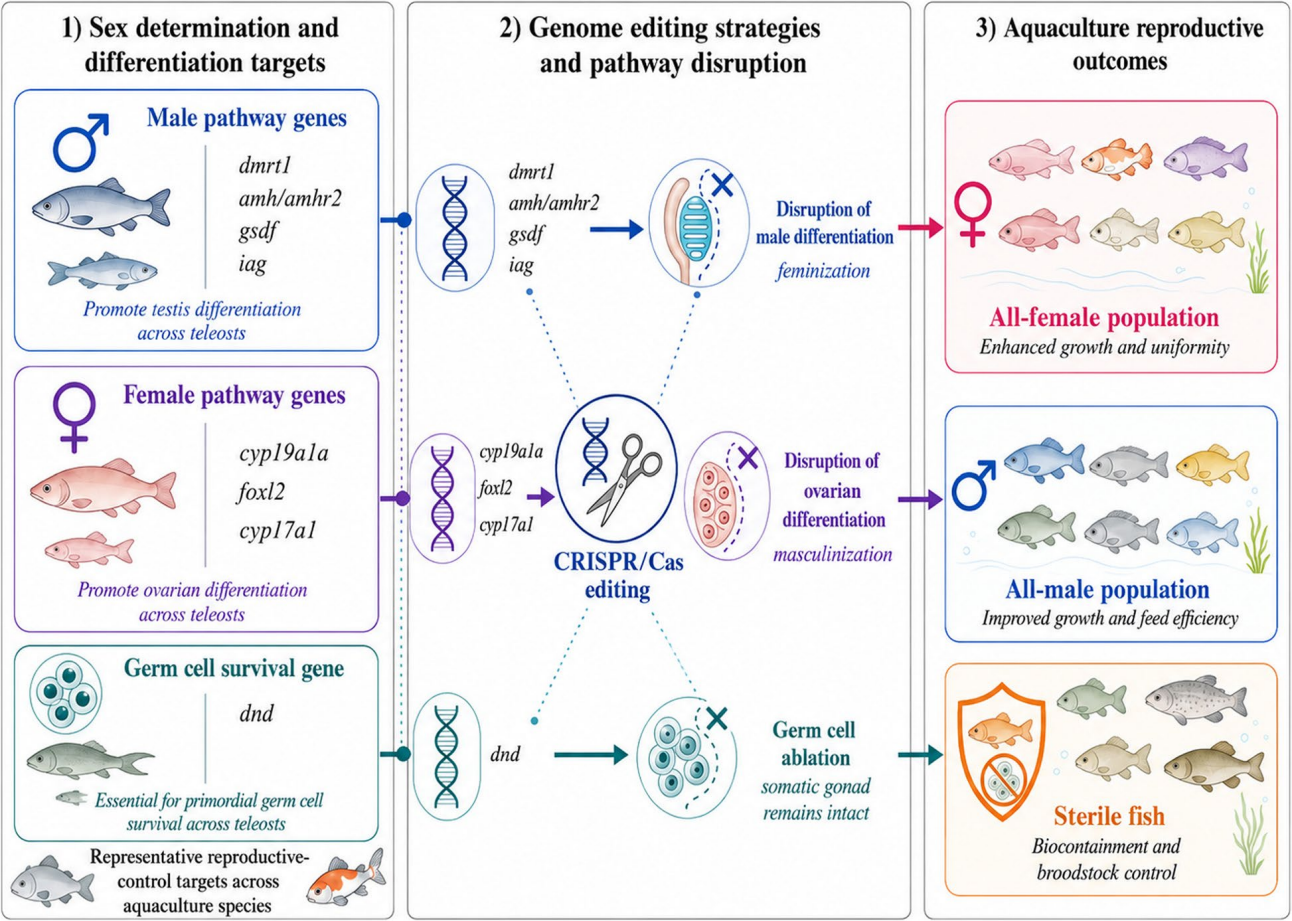
Representative genetic targets for monosex production and sterility in aquaculture species. Genes involved in sex determination, sex differentiation, and germ cell survival may be targeted by CRISPR/Cas-based genome editing to regulate reproductive outcomes in aquaculture species. Disruption of male pathway genes, such as *dmrt1*, *amh*/*amhr2*, *gsdf*, and *iag*, may impair male differentiation and promote feminization, leading to all-female populations. Conversely, disruption of female pathway genes, such as *cyp19a1a*, *foxl2*, and *cyp17a1*, may impair ovarian differentiation and promote masculinization, leading to all-male populations. Targeting germ cell survival genes, particularly dnd, can ablate endogenous germ cells while preserving the somatic gonadal environment, resulting in sterile fish. These strategies provide potential tools for monosex production, reproductive containment, and broodstock control in aquaculture

In aquaculture, CRISPR/Cas-based genome editing tools have been used to improve economic traits, including growth, disease resistance, and reproductive control (Hallerman 2021; Yang et al. 2022; Wang et al. 2025). Among these applications, the production of monosex populations has received particular attention because sex-related differences in growth rate, body size, maturation, and market value are common in many farmed aquatic species (Yang et al. 2022; Zhai et al. 2022). By targeting key genes involved in sex determination and differentiation, such as *cyp19a1a*, *foxl2*, amhy, *amhr2*, *cyp17a1*, and iag, CRISPR/Cas-mediated editing can bias sexual development toward all-male or all-female populations (Table 1; Fig. 1). This strategy has been explored in species such as tilapia, common carp, and prawns, where monosex culture can improve growth uniformity, reduce unwanted reproduction, and enhance production efficiency. In addition to monosex production, genome editing has also been used to generate sterile fish by disrupting germ cell-related genes such as *dnd*, providing a potential strategy for genetic containment and biosafety in aquaculture systems (Table 1). From a regulatory perspective, reproductive control through genome editing is especially relevant when the final edited fish does not contain foreign DNA. Small insertions, deletions, or point mutations generated by CRISPR/Cas systems may fall within SDN-1-type (Site-Directed Nuclease type 1) editing, which is often distinguished from transgenic GMO (Genetically modified organism) approaches. Base editing may further support this goal by enabling precise SNP modification without inducing double-strand breaks, making it useful for traits controlled by known nucleotide variants. However, the commercial-scale production of genome-edited monosex or sterile fish remains limited by variable editing efficiency, F_0_ mosaicism, delivery constraints, and the need to establish stable edited lines over multiple generations (Yang et al. 2022; Robinson et al. 2024; Wang et al. 2025). Across aquaculture species, CRISPR/Cas9 editing efficiency typically ranges widely from < 1% to as high as 90%, depending on species, target gene, delivery method, and gRNA design (Robinson et al. 2024; Wang et al. 2025; Yang et al. 2022). In most teleosts, biallelic mutation rates of 20–60% are commonly reported in injected embryos, whereas mosaicism frequently exceeds 40–70% in F_0_ individuals, necessitating multi-generational breeding to stabilize edited alleles. Base editors generally yield single-nucleotide conversion efficiencies of 15–50%, but their application in large-bodied farmed fish remains limited by delivery efficiency and off-target deamination (Robinson et al. 2024; Wang et al. 2025; Yang et al. 2022). Prime editing in aquatic species remains largely at the experimental stage, with efficiencies typically below 20% under current protocols (Wang et al. 2025). One major application of GE in aquaculture is the production of monosex fish by precisely manipulating genes involved in sex determination and differentiation pathways (Yang et al. 2022; Zhai et al. 2022). By targeting key sex-determining (Supplementary Table 1) and differentiation genes or regulatory elements (Table 1) using tools like CRISPR/Cas, researchers can bias development toward all-male or all-female populations (Fig. 1). This approach improves growth uniformity, reduces unwanted breeding, and enhances production efficiency in commercial aquaculture operations. However, generating many genome-edited monosex individuals currently remains challenging at commercial scale.

**Table 1:** Fish species in which monosex populations, sterility, or surrogate gamete production have been achieved using genome editing and/or germ cell transplantation

Fish species	Method	Reproductive outcome	Validated gene target(s)	Key mechanism	Application context	Reference
Zebrafish	CRISPR/Cas genome editing	Complete sterility	*dnd*	Primordial germ cell (PGC) loss due to failure of germ cell survival	Model validation of sterility strategies	(Chu et al. 2023)
Tilapia	CRISPR/Cas genome editing	All-male populations (genetic sex reversal)	*cyp19a1a*, *foxl2*, *amh*, *amhr2, dmrt1*	Disruption of ovarian differentiation pathway or enhancement of male pathway	Aquaculture production of fast-growing males	(Li et al. 2014; Liu et al. 2022)
Rainbow trout	CRISPR/Cas genome editing	Complete sterility	*dnd*	Germ cell ablation resulting in absence of gametogenesis	Genetic containment in aquaculture	(Fujihara et al. 2022)
Atlantic salmon	CRISPR/Cas genome editing	Complete sterility	*dnd*	Germ cell loss; sterile phenotype stable to adulthood	Aquaculture biocontainment	(Wargelius et al. 2016; Nishimura et al. 2024)
Atlantic salmon	Germ cell transplantation (combined with sterile host)	Surrogate gamete production	host sterility via *dnd* KO	Transplantation of donor germ cells into sterile recipient	Conservation and broodstock management	(Kleppe et al. 2025)
Common carp	CRISPR/Cas genome editing	XX neomale generation	*cyp17a1*	Disruption of male pathway	Aquaculture production of all-female cohorts	(Zhai et al. 2022)
Nibe Croaker	CRISPR/Cas genome editing	Complete sterility	*dnd*	Germ cell loss; sterile phenotype stable to adulthood	Aquaculture production of sterile fish	(Yazawa et al. 2024)
Ridgetail white prawn	CRISPR/Cas genome editing	Sex reversal/monosex populations	*iag*	Disruption of male differentiation	Aquaculture breeding applications	(Miao et al. 2023)
Giant river prawn	CRISPR/Cas genome editing	Sex reversal/monosex populations	*iag*	Disruption of male differentiation	Aquaculture breeding applications	(Wahl et al. 2023)

A complementary technology to CRISPR/Cas-based GE in producing monosex and sterile fish in aquaculture is GCT (Yoshizaki & Yazawa 2019; Okutsu et al. 2006) (Table 2), which allows researchers to transplant reproductive cells from one individual into another within a species or one species to another species. GCT offers a powerful approach for creating monosex populations and even species conservation. In fish, germ cells, including spermatogonia and oogonia, can be transplanted from a donor species into a recipient species (Yoshizaki & Lee 2018). This allows the recipient to produce offspring with the genetic makeup of the donor. This method is highly valuable in cases where donor species are endangered or difficult to breed in captivity, as it enables the conservation of genetic material and the production of donor-derived gametes in a surrogate host (Yoshizaki & Lee 2018). Comprehensive protocols for GCT have been extensively reviewed in previous studies (Lacerda et al. 2013); therefore, they are not detailed here.

**Table 2:** Genome editing (GE) and germ cell transplantation (GCT) for broodstock

Genome editing (GE) and germ cell transplantation (GCT) represent the frontier of precision aquaculture, offering solutions for producing high-performance, environmentally contained fish. GE, primarily using CRISPR/Cas systems (see Glossary), allows for the targeted modification of specific genes that control sex determination or fertility, such as the disruption of the dead end (*dnd*) gene (Fig. 1). This gene is crucial for the development and migration of primordial germ cells (PGCs), and its knockout results in fish that are incapable of reproduction, yielding highly reliable sterile fish (see Glossary). The resulting sterile fish are unable to crossbreed with wild populations, mitigating the risk of genetic introgression (see Glossary).
The second core technology is germ cell transplantation (GCT) (Fig. 1). In this technique, PGCs or spermatogonial stem cells (SSCs) are harvested from an elite donor fish and transplanted into a recipient fish (the host) that has been rendered sterile, often through the genome editing approach described above. The sterile host serves as a "surrogate." The transplanted donor cells colonize the host's gonads (see Glossary) and proceed to mature. Consequently, the sterile host fish functions as a surrogate broodstock (see Glossary), producing the eggs or sperm of the genetically superior donor species. This integrated approach allows for the secure, rapid multiplication and dissemination of desired traits in aquaculture, vastly improving biosecurity (see Glossary) and production efficiency.

**Fig. 2:**
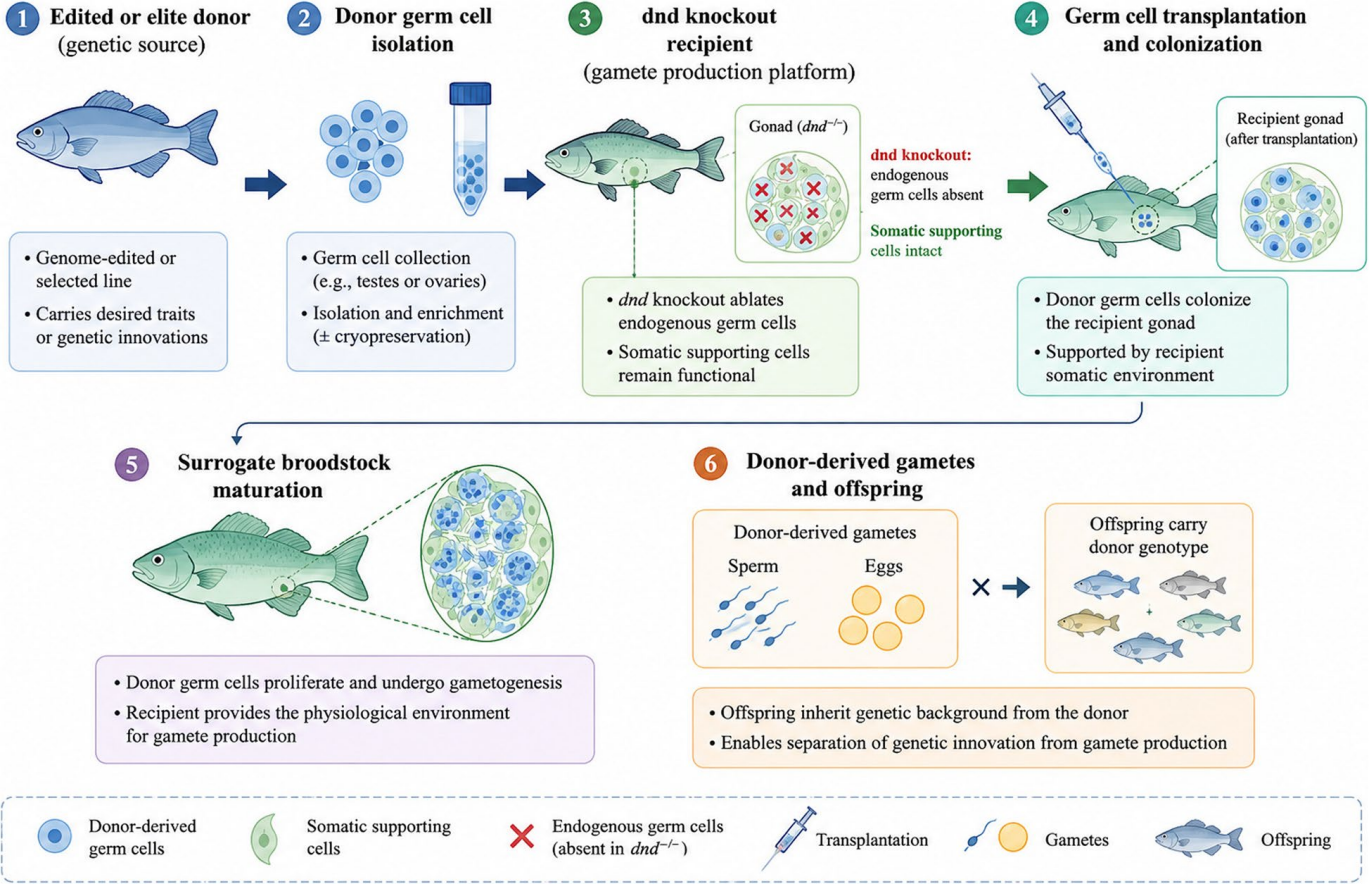
Surrogate broodstock strategy based on donor germ cell transplantation into dnd-knockout sterile recipients. Edited or elite donor fish provide donor-derived germ cells carrying desired genetic traits. These germ cells are isolated, optionally cryopreserved, and transplanted into dnd-knockout sterile recipients. In the recipients, *dnd* knockout eliminates endogenous germ cells but retains functional somatic supporting cells in the gonad. The transplanted donor germ cells colonize the recipient gonad, undergo gametogenesis, and generate donor-derived sperm or eggs. Offspring produced from these gametes carry the donor genotype, thereby decoupling genetic innovation from gamete production in surrogate broodstock systems

When combined with GE, GCT allows large-scale creation of genetically optimized, environmentally safe populations for commercial food production. For example, knockout of germline-specific genes like dead end (*dnd*) can produce sterile recipients for transplantation (Chu et al. 2023). Integration of GE and GCT into breeding programs at breeding centres can produce monosex and/or sterile fish for food (Tani et al. 2022; Fujihara et al. 2022) (Fig. 2). The principal conventional and emerging reproductive-control methods, together with their mechanisms, advantages, and limitations, are compared in Table 3.

**Table 3:** Comparison of conventional and emerging methods for producing monosex or sterile populations in finfish

Method category	Method	Main mechanism	Advantages	Limitations	References
Conventional	Hormonal sex reversal	Exogenous androgen/estrogen alters gonadal differentiation	Established; effective; scalable	Treatment-window and dose dependent; potential effects on fish physiology and the environment	(Abo-Al-Ela 2018; Huang et al. 2023; Pandian & Sheela 1995)
	Thermal manipulation	Temperature modifies sex-differentiation pathways	Simple; no genome modification	Strong species and temperature dependence; inconsistent outcomes	(Xiong et al. 2022)
	Gynogenesis/androgenesis	Uniparental genome inheritance	Produces uniparental or genetically uniform lines	Reduced survival; technically demanding; low genetic diversity	(Komen & Thorgaard 2007)
	Sex-reversal-assisted YY-supermale breeding	Sex reversal followed by genetic crossing	no hormone treatment	Requires reliable sexing markers	(Chen et al. 2019; Liu et al. 2026)
	Triploidy induction	Thermal or pressure shock induces retention of an additional chromosome set	Established; scalable in some species	Incomplete induction or sterility; species-dependent welfare and performance effects	(Benfey 2016; Piferrer et al. 2009)
Emerging	CRISPR/Cas genome editing	Targeted disruption of sex- or fertility-related genes	Precise; heritable; potentially transgene-free	Mosaicism, off-target effects, delivery and regulation	(Robinson et al. 2024; Wang et al. 2025)
	Base editing	Direct nucleotide conversion without double-strand breaks	Fewer double-strand-break-associated indels; precise nucleotide conversion	Restricted editing window and nucleotide scope; delivery and off-target deamination concerns	(Rees & Liu 2018; Wang et al. 2025)
	Prime editing	Programmable nucleotide substitution, insertion or deletion	Versatile editing without donor DNA or double-strand breaks	Low and variable efficiency in aquatic species; technically complex delivery	(Chen & Liu 2023; Wang et al. 2025)
	Germ cell transplantation	Donor germ cells colonize a sterile recipient	Germplasm conservation and propagation of elite donor lines	Limited interspecies compatibility; variable colonization efficiency; high technical and operational costs	(Fujihara et al. 2022; Kleppe et al. 2025; Yoshizaki & Yazawa 2019)
	GE combined with GCT	Gene-edited sterile recipient plus donor germ cells	Combines reproductive containment with propagation of elite donor lines	Complex multistep workflow; long validation period; uncertain regulatory pathways	(Fujihara et al. 2022; Kleppe et al. 2025; Nagasawa et al. 2019)

## Status of producing monosex and/or sterile fish via GE and GCT

### GE for monosex production

GE offers innovative approaches to produce monosex (Zhai et al. 2022) and sterile (Baloch et al. 2019) fish, improving growth efficiency and reducing ecological risks (Lu et al. 2021). Successful examples (Table 1) include:

- Common carp (*Cyprinus carpio*): CRISPR/Cas9 knockout of *cyp17a1* resulted in XX females developing as males. *cyp17a1*^+/−^ XX females showed higher body weight, demonstrating potential for all-female production (Zhai et al. 2022).
- Decapod crustaceans: In contrast to finfish, in which sex-control strategies commonly target gonadal sex-determination and differentiation genes such as *dmrt1* (Li et al. 2014; Qi et al. 2024), *cyp19a1a* (Zhang et al. 2017), *amhy* (Liu et al. 2022), and *amhr2* (Liu et al. 2022), reproductive control in decapod crustaceans has primarily focused on the androgenic gland–IAG endocrine axis. In the ridgetail white prawn (*Exopalaemon carinicauda*), *iag* knockout also caused male-to-neo-female sex reversal (Miao et al. 2023). However, CRISPR-based monosex seed production in crustaceans remains experimental because scalable embryo delivery, post-injection survival, mosaicism control, and production-scale validation remain unresolved (Miao et al. 2023; Molcho et al. 2020). By contrast, a non-GMO WW-based biotechnology in the giant freshwater prawn (*Macrobrachium rosenbergii*), based on manipulation of the androgenic gland hormone sexual switch through parental cell transfer, enabled the mass production of all-female populations and was validated in large-scale field trials over three consecutive years (Wahl et al. 2025). Thus, finfish and crustaceans share the objective of producing economically advantageous monosex populations but differ in their principal endocrine targets and current technological readiness.
- Nile tilapia (*Oreochromis niloticus*): Knockout of *dmrt1* caused XY genetic males to develop as XY females (neofemales). Crossing these neofemales with XY males produces offspring including YY individuals, which can be selected as supermales. YY supermales mated with XX females produce all-male (XY) offspring, providing a sustainable monosex production method (Liu et al. 2026). Therefore, gene editing serves as an enabling tool for generating the key founder broodstock (i.e., XY neofemales and YY supermales), whereas large-scale monosex production is achieved through conventional breeding of these edited-derived lines.

Quantitatively, in Nile tilapia, *dmrt1* knockout yields XY neofemale conversion rates of 70–100%, with subsequent YY supermale production efficiency of 18–30% (Qi et al. 2024). All-male progeny showed 10–30% higher body weight versus mixed-sex controls (Liu et al. 2026). In common carp, *cyp17a1* disruption shows 60–85% penetrance, with edited all-female cohorts growing 10–20% faster than the mixed-sex control (Zhai et al. 2022). In crustaceans, *iag* knockout feminizes 40–75% of edited males, but larval survival often drops by 20–50% (Molcho et al. 2020, 2022). Collectively, these studies demonstrate that CRISPR/Cas-mediated sex control is technically feasible across diverse aquaculture taxa. Yet, to date, no genome-edited monosex fish line has achieved routine commercial production, reflecting persistent limitations in editing efficiency, founder stabilization, and regulatory approval.

### Combination of GE and GCT for sterile and surrogate broodstock production

The integration of CRISPR/Cas GE with GCT represents a powerful and highly targeted strategy to accelerate genetic improvement in aquaculture species (Nagasawa et al. 2019; Jin et al. 2021) (Fig. 2). In this combined platform, precise genetic modifications are first introduced into donor germline cells, which are subsequently transplanted into sterile surrogate broodstock. This approach clearly separates the phase of genetic innovation from large-scale gamete production, enabling rapid dissemination of elite genotypes while maintaining strong environmental biosafety through reproductive containment. Gene editing can produce sterile fish by disrupting germ cell development. Knockout of the *dnd* gene, essential for primordial germ cell (PGC) survival, results in sterile fish (Yang et al. 2022; Güralp et al. 2020). Fertility can be rescued by transplanting donor germ cells into these sterile recipients. Successful examples include:

- Rainbow trout: Homozygous *dnd* knockout (KO) trout lack endogenous germ cells but support donor-derived germ cell development. Transplanted germ cells successfully colonized gonads and produced functional sperm and eggs, demonstrating effective surrogate broodstock technology (Fujihara et al. 2022).
- Mackerel hybrids (*Scomber* spp): Germ cell-less hybrids from female blue mackerel (*Scomber australasicus*) and male chub mackerel (*S. japonicus*) served as recipients for chub mackerel testicular cells, producing donor-derived gametes and viable offspring, showing potential for surrogate breeding (Tani et al. 2022).
- Atlantic salmon: Gonadal cell suspensions from immature salmon transplanted into mixed-sex triploid hatchlings led to functional donor-derived ovaries and sperm production in surrogates, enhancing genetic selection potential (Kleppe et al. 2025).

Knockout of *dnd* ablates germ cells and demonstrates germ cell independent gonadal differentiation in Atlantic salmon (Wargelius et al. 2016). In rainbow trout, donor-cell colonization rates reach 30–70%, with fertility restoration exceeding 60–90% in colonized recipients (Fujihara et al. 2022). Donor-derived gamete production is 80–100% of wild-type levels. Growth of *dnd* KO recipients is similar to wild-type (< 5% difference) (Fujihara et al. 2022). In Atlantic salmon, donor germ cells in triploid or *dnd*-KO recipients produce viable gametes after 3–4 years. Sterile edited salmon show equivalent growth (±3–6%), stress response, and lice resistance compared to fertile diploids, outperforming triploids which often have lower survival and higher deformity rates (Kleppe et al. 2025). Compared with conventional triploidy-based sterility, genome-editing-driven sterility provides superior biological and production performance. Editing of key fertility genes, particularly dnd and, in some species, vasa or gsdf, has produced high-efficiency reproductive confinement in experimental fish models (Jiang et al. 2022; Chu et al. 2025), with dnd disruption generating germ cell-free or sterile phenotypes. These approaches may offer a more precise alternative to triploidy, whose sterility and performance are species- and protocol-dependent. Importantly, edited sterile fish generally retain near-normal growth performance whereas triploids often exhibit reduced growth (75–90% of diploids), higher deformity rates (10–40%), and compromised welfare (Piferrer et al. 2009). Based on current evidence, genome-edited sterility is also more stable across environmental conditions, avoiding temperature- or handling-induced reversion observed in some triploid stocks (Hindar et al. 2023).

Despite these clear advantages, several technical, economic, and regulatory barriers currently restrict large-scale industrial adoption. First, editing efficiency in early embryos remains variable, and mosaicism is common, necessitating extensive founder screening and multigenerational validation to establish stable lines. Second, GCT success is highly species-specific, with wide variation in colonization, survival, and germline transmission efficiency among taxa, particularly in marine and long-lived species. Third, production timelines are prolonged in species with long generation intervals, such as salmonids or groupers, delaying commercial returns. Finally, regulatory classifications remain unclear in many regions, particularly regarding whether surrogate-produced but non-edited offspring should be regulated as genetically modified organisms.

In summary, while the integration of GE and GCT offers exceptional biological precision and long-term sustainability, the platform is still constrained by ethical and welfare considerations, cost, technical complexity, species dependence, and regulatory uncertainty, preventing it from becoming a routine industrial toolkit at present.

## Ethical and welfare considerations in producing monosex and sterile fish

Beyond chromosome-set manipulation (Komen & Thorgaard 2007), emerging genome-editing (Hallerman 2021; Yang et al. 2022; Robinson et al. 2024) and surrogate reproduction technologies (Yoshizaki & Yazawa 2019; Kleppe et al. 2025) introduce additional ethical and animal welfare considerations that warrant careful evaluation. Although CRISPR-based editing offers unprecedented precision, unintended off-target mutations, mosaicism, and developmental abnormalities may occur, particularly during the optimization stages of new editing protocols (Hallerman 2021; Yang et al. 2022; Robinson et al. 2024). Germline modifications also raise ethical questions regarding the long-term consequences of heritable genetic changes and human intervention in reproductive processes (Robinson et al. 2024). Similarly, surrogate reproduction systems often rely on sterile recipient fish, and the physiological and welfare implications of induced sterility require further investigation (Behera 2024). Public concerns surrounding reproductive engineering, animal integrity, and potential ecological risks following accidental escape may also influence societal acceptance of these technologies (Robinson et al. 2024). Consequently, rigorous welfare assessment, transparent risk evaluation, and stakeholder engagement should accompany the development and commercialization of genome-edited and surrogate-derived aquaculture stocks for producing monosex and sterile fish.

## Challenges, convergent solutions, and a roadmap to commercialization

### Persistent scientific and technical bottlenecks

Despite rapid progress, GE and GCT remain largely confined to research laboratories. Five interrelated challenges impede industrial scaling:

- Incomplete sex determination maps: In > 80% of farmed fish species, the master sex-determining gene or sex-determination pathway remains unknown (Yang et al. 2024). Without this knowledge, target selection for editing is speculative, reducing conversion efficiency and reproducibility.
- Variable editing efficiency and mosaicism: CRISPR editing in F₀ founders typically yields 20–60% biallelic mutation rates but > 50% mosaicism in teleosts (Yang et al. 2022). This necessitates multi-generational outcrossing to fix edits, which is prohibitively slow in long-generation species like salmon (3–4 years) or groupers (> 2 years).
- Low-throughput delivery: Embryo microinjection, which is the dominant delivery method, can process up to ~3,000 eggs per technician per day, with post-injection survival often < 70% (Wang et al. 2025; Yang et al. 2022). This bottleneck highlights the need for scalable delivery solutions in the roadmap, such as automation-assisted embryo handling and standardized batch-delivery platforms. Electroporation and nanoparticle delivery show promise but lack species-optimized protocols (Karp et al. 2025; Nießing et al. 2026).
- GCT species dependency: Germ cell colonization success varies widely: > 70% in rainbow trout but < 20% in many marine teleosts (Yoshizaki et al. 2011). Factors like immune rejection, gonadal niche compatibility, and donor-recipient developmental synchrony remain poorly understood.
- Regulatory and perception gaps (Table 4): Most countries lack clear frameworks for genome-edited aquatic animals (Hallerman 2021; Robinson et al. 2024; Wang et al. 2025). In regions like the EU, edited fish are regulated as GMO (Robinson et al. 2024). Even if no transgene is present, regulations may stifle investment (Robinson et al. 2024). Simultaneously, public scepticism about “gene-edited seafood” persists despite superior environmental credentials over conventional methods.China has begun to distinguish gene-edited plants from conventional GMO through specific safety evaluation guidelines, but dedicated regulations for gene-edited animals and aquatic species remain less clearly established. In the USA, genome-edited or genetically engineered animals are regulated by the FDA under a risk-based framework for heritable intentional genomic alterations, with AquAdvantage salmon serving as a key precedent. These regulatory differences, together with persistent public scepticism toward “gene-edited seafood,” highlight the need for globally harmonized, product-based governance.

**Table 4:** Regulatory divergence in genome-edited aquaculture and outstanding questions on genome-edited monosex and sterile fish for commercial production

The global regulatory landscape for genome-edited fish remains fragmented, creating uncertainty for research and investment. The European Union regulates all genome-edited organisms as genetically modified organisms (GMO), requiring full risk assessment and traceability—even when no foreign DNA is present. In contrast, Argentina, Brazil, Japan, and the UK have adopted product-based approaches: if the final organism could have arisen through conventional breeding or natural mutation (e.g., a diploid sterile fish with a point mutation in *dnd*), it is not classified as a GMO. This divergence impedes international collaboration and trade. Harmonization under frameworks like the Codex Alimentarius or the Organisation for Economic Co-operation and Development (OECD) Consensus Documents could accelerate the adoption of environmentally beneficial technologies while ensuring biosafety and consumer transparency.
Outstanding questions
Despite significant progress, several key questions must be addressed before genome-edited monosex, and sterile fish achieve commercial viability:
• How can genome editing efficiency (specifically reducing mosaicism and increasing HDR success) be improved in fish embryos and germline stem cells (GSCs) to achieve the high throughput and reliability needed for industrial-scale production?
• What are the most effective non-invasive or high-throughput delivery systems (e.g., viral, lipid-based, or electroporation methods) for introducing CRISPR components into large quantities of fish eggs or GSCs without compromising viability or reproductive function?
• How will regulatory agencies in major aquaculture markets (e.g., EU, US, China) classify the resulting sterile or monosex fish (e.g., as GMO or non-GMO), and what standardized protocols are needed to facilitate global commercialization?
• Can the cost-effectiveness of the integrated genome editing and GSC transplantation platform be reduced to a competitive level (e.g., within 1.5-2x of conventional methods) by optimizing donor cell proliferation and transplantation success rates?
• What are the long-term ecological and evolutionary consequences of introducing sterile fish into aquatic ecosystems, and how can safety-by-design strategies be further validated to ensure environmental containment?
• How does targeted gene knockout or disruption of fertility genes (e.g., *dnd*, *gsdf*) affect the overall fitness, health, and meat quality of the resulting sterile broodstock compared to traditional methods like triploidy?
• What public engagement and communication strategies are most effective in building consumer trust and acceptance for food products derived from genome-edited fish, particularly regarding sterile or monosex populations?
• Can universal surrogate broodstock protocols be developed that allow a single, easily cultured host species to reliably produce gametes for multiple, commercially important target species, thereby simplifying hatchery operations globally?
• What is the comparative cost per juvenile for genome-edited sterile fish vs. triploids vs. hormone-treated monosex fish at commercial production scales (≥1 million juveniles/year)?
• How can small-scale and resource-limited farmers access genome-edited broodstock without being locked into proprietary contracts that undermine local breeding programs?

### Environmental effects on reproductive-control outcomes

Environmental conditions can influence sex differentiation, treatment efficacy, and the subsequent performance of monosex or sterile populations. Temperature is the best-characterized environmental influence on sex determination and differentiation in teleosts, although hypoxia, stocking density, pH, and social conditions may also affect sexual development in some species (Rajendiran et al. 2021). During the thermosensitive period, elevated temperature can activate the stress axis and increase cortisol production. Cortisol may promote masculinization by suppressing gonadal aromatase (*cyp19a1a*) and estrogen synthesis and by activating male-pathway genes such as *amh* (Goikoetxea et al. 2017). However, these responses are species-, strain-, and stage-dependent. This variation explains the inconsistent outcomes of thermal manipulation and may also alter the developmental window during which hormonal sex reversal is effective (Huang et al. 2023; Xiong et al. 2022).

For genetic and chromosome-set approaches, environmental conditions do not change the induced genotype or ploidy status but may affect the expression of the intended phenotype, survival, growth, and welfare. Large triploid Atlantic salmon showed reduced aerobic scope and lower tolerance to heat and hypoxia, consistent with oxygen-supply limitations associated with larger cell size and lower gill lamellar density (Hvas et al. 2026). Therefore, reproductive-control technologies should be validated across representative farming environments, with temperature, dissolved oxygen, sex ratio, fertility, survival, growth, and welfare reported as core performance indicators.

### Convergent technological and policy solutions

Overcoming these barriers requires more than incremental biology—it demands convergence across disciplines:

- AI-augmented target discovery: Machine learning models trained on comparative genomics, ATAC-seq, and single-cell gonad atlases can predict sex/fertility genes in non-model species, accelerating target validation (Yang et al. 2024; Garcia-Alonso et al. 2022; Kelley 2020). For example, comparative genomics can be used to identify conserved orthologs of known sex-determining or germ-cell genes from model species, while gene-expression and co-expression networks can prioritize candidates that are specifically active during gonadal differentiation. In addition, ATAC-seq and single-cell transcriptomic data can help locate active regulatory elements and cell-type-specific markers in testes, ovaries, or primordial germ cells, allowing machine learning models to rank candidate coding genes and regulatory regions for GE. These approaches are especially useful in non-model species where functional annotation is incomplete and experimental validation is slow. However, their accuracy depends on high-quality, species-specific multi-omics databases, which remain limited for many aquaculture species, particularly for cutting-edge resources such as single-cell gonad atlases and chromatin accessibility maps. To date, this approach remains conceptual for farmed fish, requiring species-specific training data that are not yet available for most taxa.
- Automated embryo editing platforms: Robotic microinjection arms (Nießing et al. 2026), microfluidic embryo arrays (Diouf et al. 2025), and CRISPR ribonucleoprotein (RNP) electroporation (Karp et al. 2025) may scale editing to > 50,000 embryos/day, which is essential for high-fecundity species like tilapia (1,000–2,000 eggs/female). These platforms directly address the low-throughput delivery bottleneck by reducing manual embryo handling, standardizing reagent delivery, and enabling parallel processing of large embryo batches.
- Digital phenotyping for screening: Non-invasive imaging and AI-based behavior/growth tracking enable rapid identification of monosex or sterile candidates without routine dissection (Fu & Yuna 2022). For example, image-based models could be used to monitor growth uniformity, gonadal development-related morphology, or abnormal developmental phenotypes, thereby reducing culling and accelerating line development.
- Cryobanked germ cell networks: Global biobanks of cryopreserved spermatogonia/oogonia, paired with cloud-based genetic metadata, could enable “germplasm-on-demand,” where elite donor cells are shipped and transplanted into locally adapted sterile surrogates (Yoshizaki & Lee 2018; Wylie et al. 2025). When integrated with genomic records and digital breeding platforms, such networks could support more efficient matching of donor germ cells, recipient lines, and production environments.
- Harmonized “product-based” regulation: Regulatory frameworks should focus on the final trait (e.g., sterility, monosex) rather than the process (editing vs. triploidy). Countries like Argentina, Brazil, and Japan have pioneered such approaches for crops and fish—offering models for international alignment (Hallerman et al. 2024).

### A quantitative roadmap to industrial adoption

**Fig. 3:**
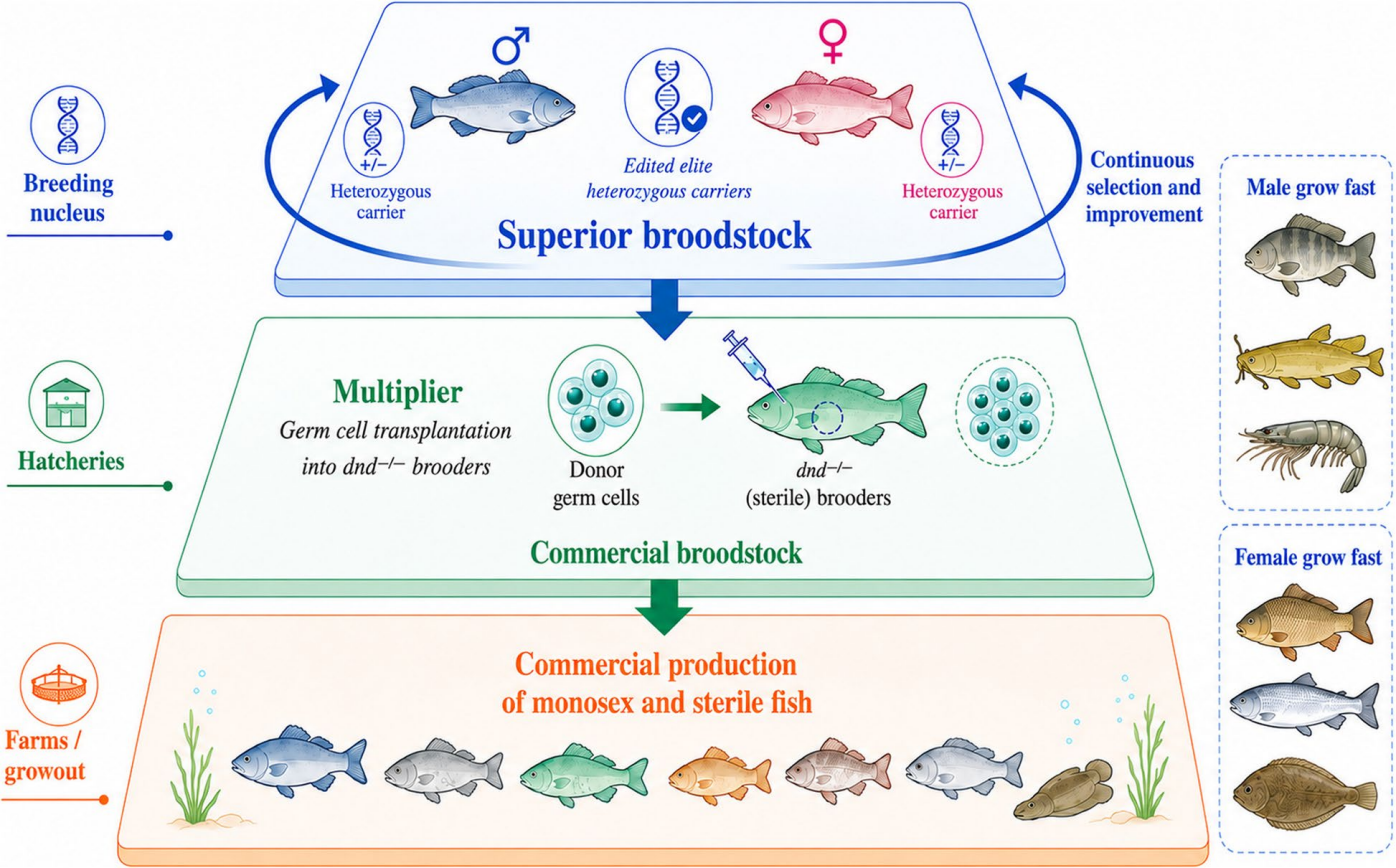
A scalable two-tier platform for producing monosex and sterile fish in commercial aquaculture. A two-tier system separates genetic innovation from large-scale gamete production to enable scalable and biocontained aquaculture. Tier 1 **–** Genetic innovation (breeding center)**:** Precise genome editing is performed to generate stable edited lines. Target genes may include those involved in sex determination or germ cell development, such as c*yp19a1a*, *cyp17a1*, *gsdf*, *dmrt1*, or *dnd*. The resulting lines are maintained as elite heterozygous carriers and used to produce homozygous mutants for monosex or sterility applications. For sterility applications, biallelic disruption of germline-essential genes (e.g., *dnd*) produces individuals lacking endogenous germ cells. For monosex production, edits in key sex-determining or differentiation genes bias sexual development toward all-male or all-female phenotypes. Stable edited founders are established through breeding and molecular validation. Tier 2 – Surrogate gamete production (hatchery level)**:** Sterile recipients serve as surrogate broodstock. Donor germ cells from genetically superior or sex-controlled lines are transplanted into these recipients. The transplanted cells colonize the gonads and generate functional gametes derived exclusively from the donor genome. This enables large-scale multiplication of elite genotypes while maintaining reproductive containment of the surrogate host. At the farm level, production populations consist of monosex and/or sterile fish derived from controlled breeding pipelines. This framework is particularly suited for high-fecundity species and species exhibiting strong sexual size dimorphism (e.g., Nile tilapia, yellow catfish, freshwater prawn, common carp, Atlantic salmon, half-smooth tongue sole). By decoupling genome editing from commercial seed production, this integrated system enhances biosafety, genetic dissemination speed, and scalability for sustainable aquaculture

For genome-edited monosex and sterile fish to achieve broad commercial use (Fig. 3) within the next decades, the field must meet five performance benchmarks (Table 5). Achieving these thresholds will require coordinated investment in species-specific toolkits, open-access germplasm repositories, and public–private partnerships. Critically, success will hinge not only on biological performance but on co-developing trust: transparent labelling, third-party environmental risk assessments, and farmer/consumer co-design of deployment models. Only when biology, engineering, economics, and ethics advance in lockstep will precision reproductive control transition from a laboratory marvel to a pillar of sustainable food systems.

**Table 5:** Commercialization benchmarks for genome-editing and germ cell transplantation for aquaculture production*

Benchmark	Target threshold	Status
Founder editing efficiency	> 60%	0.2–90% (species-dependent)
Mosaicism in F₀	< 10%	Typically, 40–70%
Germ cell colonization	> 70%	20–70% (highly variable)
Fertility restoration in surrogates	> 90%	60–90% (in trout/salmon)
Growth vs. diploid controls	≥ 95%	~ 100% for *dnd* KO; < 90% for triploids

^*^These represent the authors' own perspective, not official positions. The benchmarks were derived based on a literature survey or expert consensus

## Summary

The quest for monosex and sterile fish is no longer confined to the domain of endocrinology or polyploidy. It has entered an era of precision reproductive biotechnology. CRISPR-based GE and GCT now offer heritable, scalable, and ecologically responsible alternatives to conventional methods, with demonstrated success across finfish and crustaceans. These tools do more than accelerate growth or prevent genetic leakage; they represent a paradigm shift toward programmable aquaculture, where reproduction is not left to chance but engineered for sustainability, resilience, and containment.

Yet technological prowess alone is insufficient. The path from laboratory proof-of-concept to global adoption is paved with interlocking challenges (see Outstanding questions in Table 4): incomplete knowledge of sex-determination networks in non-model species, mosaicism and delivery bottlenecks, species-specific variability in germ cell colonization, and perhaps most critically fragmented regulatory landscapes and public scepticism. Overcoming these will require more than better editors or surrogates; it demands convergence between molecular biologists, automation engineers, data scientists, policymakers, and farmers.

Although many questions remain to be answered (see Outstanding questions in Table 4), the ultimate measure of success will not be editing efficiency or colonization rates, but whether these innovations deliver safe, affordable, and environmentally sound seafood without compromising biodiversity or equity. If the field can align biological precision with social responsibility, establishing transparent governance, equitable access to germplasm, and robust biocontainment, then genome-edited monosex and sterile fish could become cornerstones of a climate-adaptive, circular aquaculture economy.

As genome editing and surrogate breeding technologies advance toward commercial deployment for monosex and sterile fish production, integrating animal welfare, ethical governance, and societal acceptance into breeding programs will be as important as achieving genetic and production gains.

## Supplementary Information

Supplementary Material 1. Key genes and major QTL for sex determination in important aquaculture fish species.

## Data Availability

No new datasets were generated for this review.
