# Supplementary material for: Engineering monosex and sterile fish for food production: from conventional methods to CRISPR-based precision
Source: Adv Biotechnol (Singap). 2026 Aug 4;4(3):30. doi: 10.1007/s44307-026-00128-5 (PMC13438497; doi:10.1007/s44307-026-00128-5)
Supplement: Supplementary file 1 [file 44307_2026_128_MOESM1_ESM.docx]

**Supplementary Table** **1** Key genes and major QTL determining sex in important aquaculture fish species.

| Species | Sex chromosomes | Sex-determining genes/QTL | Reference |
| --- | --- | --- | --- |
| Pufferfish (*Takifugu rubripes)* | XY | *Amhr2* | [1] |
| Nile tilapia (*Oreochromis niloticus*) | XY | *AmahY* | [2,3] |
| Luzon ricefish (*O. luzonensis*) | XY | *gsdf* | [4] |
| Rainbow trout (*O. mykiss*) | XY | *sdY* | [5,6] |
| Atlantic cod (*Gadus morhua*) | XY | *zkY* | [7] |
| Patagonian pejerrey (*Odontesthes hatcheri*) | XY | *AmahY* | [4] |
| Spotbanded scat (*Selenotoca multifasciata*) | XY | *Dmrt1Y* | [8] |
| longsnout catfish (*Leiocassis longirostris*) | XY | *ptf1aY* | [9] |
| channel catfish (*Ictalurus punctatus*) | XY | *BCAR1* | [10] |
| Hybrid tilapia (*Oreochromis spp.* x *O. mossambicus*) | XY or ZW | Two QTL on LG1 & 22, LG3, LG14 | [11] |
| Atlantic halibut (*H. hippoglossus*) | XY | A major QTL on LG13 | [12] |
| Sea Bream (*Sparus aurata* L.) | XY | Two sex-linked QTL on LG21 | [13] |
| European seabass *(Dicentrarchus labrax* L) | XY | Several QTL on LG6, 11,12,18-21 | [14] |
| *White Shrimp (Litopeneaus vannamei)* | ZW | A major QTL on LG18 | [15] |
| Tiger shrimp (*Penaeus monodon*) | ZW | A major QTL on LG30 | [16,17] |
| Mandarin fish (*Siniperca chuatsi*) | XY | A major QTL on LG23 | [18] |
| Yellow drum (*Nibea albiflora*) | XY | A major QTL on LG9, dmrt1 | [19,20] |
| Bighead carp (*Hypophthalmichthys* *nobilis*) | XY | Two QTL on L19 & 21 | [21] |
| Common carp (*Cyprinus carpio*) | XY | Seven QTL on seven LGs | [22,23] |
| Swimming Crab (Portunus trituberculatus) | XY | A major QTL on LG24 | [24] |
| Mitten crab (*Eriocheir sinensis*) | ZW | A major QTL on LG60 | [25] |
| Half-smooth tongue-sole (*Cynoglossus semilaevis*). | ZW | Several QTL on different LGs | [26] |
| Mud Crab (*Scylla paramamosain*) | ZW | Two QTL on LG32 | [27] |
| Channel catfish (*Ictalurus punctatus*) | XY | A major QTL on LG17 | [28] |
| Snakehead (*Channa argus*) | XY | A major QTL on LG16 | [29], [30] |
| bighead carp (*Hypophthalmichthys nobilis*) | XY | A major QTL | [31] |
| Silver carp (*Hypophthalmichthys molitrix*) | XY | A major QTL | [31] |
| Largemouth bass (*Micropterus salmoides*) | XY | 13 QTL on six LGs | [32] |
| Large-ccale loach (*Paramisgurnus* *dabryanus*) | ZW | Two QTL on LG 9 & 11 | [33] |
| Yellowtail (*Seriola quinqueradiata*) | ZW | Two QTL in LG12 hsd17b1 | [34] |
| Turbot (*Scophthalmus maximus*) | ZW | Four QTL on LG5, 6, 8 & 21 | [35] |

**References**

1.	Kamiya, T. *et al.* (2012) A trans-species missense SNP in Amhr2 is associated with sex determination in the tiger pufferfish, *Takifugu rubripes* (fugu). *PLoS Genet.* 8, e1002798.

2.	Li, M.H. *et al.* (2015) A tandem duplicate of anti-mullerian hormone with a missense SNP on the Y chromosome is essential for male sex determination in Nile tilapia, *Oreochromis niloticus*. *PLoS Genet.* 11, e1005678.

3.	Jiang, D.N. *et al.* (2016) gsdf Is a downstream gene of dmrt1 that functions in the male sex determination pathway of the Nile tilapia. *Mol. Reprod. Dev.* 83, 497-508.

4.	Hattori, R.S. *et al.* (2013) Genotypic sex determination in teleosts: insights from the testis-determining amhy gene. *Gen. Comp. Endocrinol.* 192, 55-59.

5.	Yano, A. *et al.* (2012) An immune-related gene evolved into the master sex-determining gene in rainbow trout, *Oncorhynchus mykiss*. *Curr. Biol.* 22, 1423-1428.

6.	Yano, A. *et al.* (2013) The sexually dimorphic on the Y‐chromosome gene (sdY) is a conserved male‐specific Y‐chromosome sequence in many salmonids. *Evol. Appl.* 6, 486-496.

7.	Kirubakaran, T.G. *et al.* (2018) A zinc knuckle gene on the Y chromosome (zkY) determines sex in Atlantic cod (*Gadus morhua*). *bioRxiv*, 362376.

8.	Jiang, D.-N. *et al.* (2022) Establishment of the Y-linked Dmrt1Y as the candidate sex determination gene in spotbanded scat (*Selenotoca multifasciata*). *Aquac. Rep.* 23, 101085.

9.	Ye, H. *et al.* (2023) Identification of a candidate sex-determining gene, ptf1aY, in the Chinese longsnout catfish (*Leiocassis longirostris*) through high-throughput sequencing. *Aquac. Rep.* 32, 101730.

10.	Bao, L. *et al.* (2019) The Y chromosome sequence of the channel catfish suggests novel sex determination mechanisms in teleost fish. *BMC Biol.* 17, 6.

11.	Lin, G. *et al.* (2016) Mapping QTL for sex and growth traits in salt-tolerant tilapia *(Oreochromis spp*. X *O. mossambicus*). *PLoS ONE* 11.

12.	Palaiokostas, C. *et al.* (2013) Mapping the sex determination locus in the Atlantic halibut (*Hippoglossus hippoglossus*) using RAD sequencing. *BMC Genom.* 14.

13.	Loukovitis, D. *et al.* (2011) Quantitative trait loci involved in sex determination and body growth in the gilthead sea bream (*Sparus aurata* L.) through targeted genome scan. *PLoS ONE* 6, e16599.

14.	Palaiokostas, C. *et al.* (2015) A new SNP-based vision of the genetics of sex determination in European sea bass (*Dicentrarchus labrax*). *Genet.. Sel. Evol.* 47, 68.

15.	Yu, Y. *et al.* (2017) Identification of sex-determining loci in Pacific white shrimp *Litopeneaus vanname*i using linkage and association analysis. *Mar. Biotechnol.* 19, 277-286.

16.	Robinson, N.A. *et al.* (2014) QTL for white spot syndrome virus resistance and the sex-determining locus in the Indian black tiger shrimp (*Penaeus monodon*). *BMC Genom.* 15, 731.

17.	Guo, L. *et al.* (2019) A high-density genetic linkage map and QTL mapping for sex in black tiger shrimp (*Penaeus monodon*). *Front. Genet.* 10, 326.

18.	Sun, C.F. *et al.* (2017) Construction of a high-density linkage map and mapping of sex determination and growth-related loci in the mandarin fish (*Siniperca chuatsi*). *BMC Genom.* 18, 446.

19.	Qiu, C. *et al.* (2018) A high-density genetic linkage map and QTL mapping for growth and sex of yellow drum (*Nibea albiflora*). *Sci. Rep.* 8, 1-12.

20.	Sun, S. *et al.* (2023) Study on sex-linked region and sex determination candidate gene using a high-quality genome assembly in yellow drum. *Aquaculture* 563, 738987.

21.	Zhou, Y. *et al.* (2020) QTL fine mapping for sex determination region in bighead carp (*Hypophthalmichthys nobilis*) and comparison with silver carp (*Hypophthalmichthys molitrix*). *Mar. Biotechnol.* 22, 41-53.

22.	Peng, W. *et al.* (2016) An ultra-high density linkage map and QTL mapping for sex and growth-related traits of common carp (*Cyprinus carpio*). *Sci. Rep.* 6, 1-16.

23.	Feng, X. *et al.* (2018) A high-resolution genetic linkage map and QTL fine mapping for growth-related traits and sex in the Yangtze River common carp (*Cyprinus carpio* haematopterus). *BMC Genom.* 19, 230.

24.	Lv, J. *et al.* (2018) QTL mapping and marker identification for sex-determining: indicating xy sex determination system in the swimming crab (*Portunus trituberculatus*). *Front. Genet.* 9, 337.

25.	Cui, Z. *et al.* (2015) High-density linkage mapping aided by transcriptomics documents ZW sex determination system in the Chinese mitten crab *Eriocheir sinensis*. *Heredity* 115, 206-215.

26.	Wang, J. *et al.* (2015) QTL interaction of sex determination in half-smooth tongue-sole (*Cynoglossus semilaevis*). *Journal of Agricultural Biotechnology* 23, 89-95.

27.	Waiho, K. *et al.* (2019) High-density genetic linkage maps provide novel insights into ZW/ZZ sex determination system and growth performance in mud crab (*Scylla paramamosain*). *Front. Genet.* 10, 298.

28.	Zhang, S. *et al.* (2019) Construction of a high-density linkage map and QTL fine mapping for growth-and sex-related traits in channel catfish (*Ictalurus punctatus*). *Front. Genet.* 10, 251.

29.	Liu, H. *et al.* (2020) High-density genetic linkage map and QTL fine mapping of growth and sex in snakehead (*Channa argus*). *Aquaculture* 519, 734760.

30.	Wang, L. *et al.* (2019) Constructing high-density genetic maps and developing sexing markers in northern snakehead (*Channa argus*). *Mar. Biotechnol.* 21, 348-358.

31.	Liu, H. *et al.* (2018) Sex-specific markers developed by next-generation sequencing confirmed an XX/XY sex determination system in bighead carp (*Hypophthalmichthys nobilis*) and silver carp (*Hypophthalmichthys molitrix*). *DNA Res.* 25, 257-264.

32.	Zhu, C. *et al.* (2019) Construction of a high-density genetic linkage map and QTL mapping for growth traits in *Pseudobagrus ussuriensis*. *Aquaculture* 511, 734213.

33.	Wei, J. *et al.* (2019) A high-density genetic linkage map and qtl mapping for sex and growth-related traits of large-scale loach (*Paramisgurnus dabryanus*). *Front. Genet.* 10, 1023.

34.	Fuji, K. *et al.* (2010) Identification of the sex-linked locus in yellowtail, Seriola quinqueradiata. *Aquaculture* 308, S51-S55.

35.	Vinas, A. *et al.* (2012) Mapping of DNA sex-specific markers and genes related to sex differentiation in turbot (*Scophthalmus maximus*). *Mar. Biotechnol.* 14, 655-663.
